# Sustainable Upcycling of Fisheries and Aquaculture Wastes Using Fish-Derived Cold-Adapted Proteases

**DOI:** 10.3389/fnut.2022.875697

**Published:** 2022-04-07

**Authors:** Zied Khiari

**Affiliations:** National Research Council Canada, Aquatic and Crop Resource Development Research Centre, Halifax, NS, Canada

**Keywords:** seafood waste, cold-adapted enzymes, proteases, upcycling, sustainable valorization processes

## Abstract

The fisheries and aquaculture industries are some of the major economic sectors in the world. However, these industries generate significant amounts of wastes that need to be properly managed to avoid serious health and environmental issues. Recent advances in marine waste valorization indicate that fish waste biomass represents an abundant source of high-value biomolecules including enzymes, functional proteins, bioactive peptides, and omega-3 rich oils. Enzyme-assisted processes, for the recovery of these value-added biomolecules, have gained interest over chemical-based processes due to their cost-effectiveness as well as their green and eco-friendly aspects. Currently, the majority of commercially available proteases that are used to recover value-added compounds from fisheries and aquaculture wastes are mesophilic and/or thermophilic that require significant energy input and can lead to unfavorable reactions (i.e., oxidation). Cold-adapted proteases extracted from cold-water fish species, on the other hand, are active at low temperatures but unstable at higher temperatures which makes them interesting from both environmental and economic points of view by upcycling fish waste as well as by offering substantial energy savings. This review provides a general overview of cold-adapted proteolytic enzymes from cold-water fish species and highlights the opportunities they offer in the valorization of fisheries and aquaculture wastes.

## Introduction

The fisheries and aquaculture industries represent some of the major economic sectors in the world. A recent report by the Food and Agricultural Organization (FAO) indicated that the total world fisheries and aquaculture production (fish, crustaceans, and mollusks) reached 177.8 million tons in 2019 and generated USD 406 billion in terms of total first sale value (1). However, 50–70% of this global fisheries and aquaculture production is regarded as waste and is discarded either at sea or in landfills (2–4).

Organic wastes generated from food, aquaculture and fisheries industries represent a serious global issue due to their economic, social and environmental impacts (5). In addition, these wastes can, to a certain degree, contribute to climate change through greenhouse gas (GHG) emissions when dumped in landfills. For instance, according to a report by Bogner et al. (6), methane emission from landfills and wastewater jointly accounted for nearly 90% of waste sector emissions, which translated to about 18% of annual global anthropogenic methane emissions (6). Waste valorization, as a mitigation approach, has a positive impact on climate change, which is mainly attributable to the reduction in GHG emissions due to recycling and waste minimization as well as energy recovery from the waste (7). Several studies indicated that fisheries and aquaculture wastes can be considered as potential sources of biomolecules including enzymes, functional proteins, bioactive peptides, omega-3 rich oils and polysaccharides such as chitin (8–12). Current industrial processes for the extraction of these biomolecules are unsustainable as they are heavily based on the use of harsh chemical treatments and/or costly enzymatic reactions which consume significant amounts of water and energy and generate large volumes of effluent that needs treatment before discharge (13, 14). The majority of commercially available enzymes that are used to recover value-added compounds from fish waste have optimal activities between 50 and 80°C. This temperature range poses a serious safety risk (i.e., growth of pathogenic micro-organisms) and unfavorable reactions (i.e., oxidation). By contrast, when used at lower temperatures, their enzyme activity is dramatically reduced. Cold-adapted enzymes, on the other hand, are active between 0 and 30°C but unstable at temperatures higher than 50°C (15).

Fish viscera represent an abundant by-product of fisheries and aquaculture industries and typically account for 5–10% of the fish body weight (16). Fish viscera comprise a number of digestive enzymes including acidic (i.e., pepsin) and alkaline proteases (i.e., trypsin, chymotrypsin, and elastase) that are widely used in bioprocessing (16). In addition, proteases present in the viscera of cold-water fish species have been reported to possess cold-adapted properties (17–19). Therefore, using fish-derived cold-adapted proteases to upcycle fisheries and aquaculture wastes can represent a sustainable approach for the conversion of wastes into value-added products which would utilize the wastes completely and eliminate their negative environmental impact. The aim of this review is to provide a general overview of cold-adapted proteolytic enzymes from cold-water fish species and to highlight the opportunities they offer in the valorization of fisheries and aquaculture wastes.

## Enzyme Cold Adaptation

The mechanisms of cold adaptation are complex; however, one common characteristic of all cold-adapted enzymes is their high molecular flexibility compared with their mesophilic and thermophilic counterparts, which show low activity and are structurally rigid at low temperatures (20–22). The increased molecular flexibility, which is required to compensate for the low working temperature, has been proposed as the main structural feature of cold-adapted enzymes (23) and is currently the most widely accepted theory for cold adaptation (24). However, there is a debate whether the flexibility of cold-adapted enzymes is a global flexibility (i.e., flexibility throughout the enzyme structure) or a local flexibility (i.e., distinct regions in the enzyme structure) (22). Other features observed with cold-adapted enzymes include (i) a smaller number of hydrogen bonds, (ii) structures that are less densely packed, (iii) higher surface hydrophilicity, as well as (iv) an increased number of methionine residues (21).

Current evidences also indicate that the high specific activity of cold-adapted enzymes is most of the time associated with a low thermo-stability (23). In this regard and compared to mesophilic and thermophilic homologs, cold-adapted enzymes have a lower thermal stability (in order to prevent freezing) and exhibit a lower enthalpy and a more negative entropy of activation (25). Additionally, several cold-adapted proteases have been reported to be more susceptible to auto-digestion compared to their mesophilic analogs – a feature that has been associated with their increased catalytic efficiency and decreased thermal stability (21, 26).

## Cold-Adapted Proteases From Cold-Water Fish Species

Proteases, the enzymes that catalyze the hydrolysis of peptide bonds, are the major group of industrial enzymes, accounting for more than 60% of the global enzyme market (27, 28). Proteases are divided into endopeptidases and exopeptidases depending on their cleavage points within the peptide chain. Based on the reactive groups at the active site, proteases can further be categorized into serine-, cysteine-, aspartic-, or metallo-peptidases. So far, serine peptidases are the most extensively investigated and characterized protease subgroup (29).

To date, little research has been conducted on fish-derived cold-adapted proteolytic enzymes. Perhaps, the sensitivity of cold-adapted enzymes to autolysis, thermal inactivation as well as molecular aggregation could explain the limited studies in this area (21). The available studies on cold-adapted fish derived proteolytic enzymes mainly focused on a small number of acidic (i.e., pepsin) and alkaline (i.e., trypsin, chymotrypsin, and elastase) proteases. These acidic and alkaline proteolytic enzymes, which represent about 5% of the total fish mass (28), are the major digestive proteolytic enzymes in fish viscera and are found in the stomach and pyloric ceca/small intestine, respectively.

### Acidic Proteases

Pepsin, which belongs to the aspartic endopeptidase family (Table 1), is the major acidic protease in fisheries and aquaculture wastes. Pepsin has been extracted and characterized from a number of cold-water fish species (Table 1), including Arctic capelin (18), Greenland cod (30), Polar cod (19), Atlantic cod (18, 31, 32), Chum salmon (33), and rainbow trout (34, 35).

**TABLE 1:** General characteristics of major digestive proteases present in fisheries and aquaculture wastes.

	Pepsin	Trypsin	Chymotrypsin	Elastase
Enzyme Commission number	3.4.23.1	3.4.21.4	3.4.21.1	3.4.21.36
Chemical Abstracts Service number	9001-75-6	9002-07-7	9004-07-3	848900-32-3
Classification	Aspartic endopeptidase	Serine endopeptidase	Serine endopeptidase	Serine endopeptidase
Molecular weight	34.5 kDa	23.3 kDa	25.6 kDa	26.0 kDa
Optimum pH range	2.0–3.5	7.5–8.5	7.8–8.0	8.5
Active site residues	Aspartic acid (D32) Aspartic acid (D215)	Histidine (H63) Aspartic acid (D107) Serine (S200)	Histidine (H57) Aspartic acid (D102) Serine (S195)	Histidine (H71) Aspartic acid (D119) Serine (S214)
Specificity	Preferentially cleaves C-terminal of leucine as well as phenylalanine, and to a lesser degree glutamic acid linkages	Catalyzes the hydrolysis of peptides on the C-terminal side of lysine and arginine residues	Preferentially hydrolyzes peptide bonds involving tyrosine, tryptophan, and phenylalanine	Catalyzes the cleavage of carboxyl groups present on glycine, valine, and alanine
Examples of cold-water fish species source of cold-adapted proteases	Arctic capelin, Greenland cod, Polar cod, Atlantic cod, Chum salmon, and rainbow trout	Atlantic cod, rainbow trout, anchovy, Monterey sardine, Coho salmon, and Atlantic salmon	Atlantic cod, rainbow trout, anchovy, and Monterey sardine	Atlantic cod

*Adapted from BRENDA (The Comprehensive Enzyme Information System) and Worthington Enzyme Manual (37).*

Fish-derived pepsin has been utilized in various applications including protein hydrolysis, collagen extraction and as a rennet substitute in addition to being useful for some therapeutic purposes (36). The use of fish-derived cold-adapted pepsin to treat fisheries and aquaculture wastes could represent a low-cost and safe waste treatment approach since processing the organic biomass at low temperatures avoids possible side effects (i.e., oxidation) while the low pH conditions reduce the risk of spoilage as acidic pH inhibits most pathogens (14).

### Alkaline Proteases

Trypsin, chymotrypsin, and elastase, which all belong to the serine-protease family, are the main alkaline proteases in fisheries and aquaculture wastes. The general characteristics of these three proteases are summarized in Table 1.

Cold-adapted serine proteases have been isolated and characterized from a number of cold-water fish species (Table 1). For example, cold-adapted trypsin, chymotrypsin and elastase were extracted from Atlantic cod (38–44). Trypsin and chymotrypsin were isolated from rainbow trout (45, 46) as well as anchovy (47–49). Other studies focused on the recovery of trypsin and chymotrypsin from Monterey sardine (50–54). When it comes to salmonids, trypsin with cold-adapted properties was extracted from Coho salmon (55) and Atlantic salmon (56–58).

These three serine proteases (trypsin, chymotrypsin, and elastase), which are found in the pancreatic juices and are highly active in the pyloric ceca and small intestine of fish, have been used in several industrial applications due to their high specificity as well as stability and activity under severe unfavorable conditions, such as alkaline pH and in the presence of surfactants or oxidizing agents (59). Some examples of industrial applications of cold-adapted alkaline proteases comprise the production of a seafood flavoring hydrolyzate from crustaceans and as a key ingredient in natural skin care products (21) in addition to being applied in biomedical research (60).

## Typical Enzyme Recovery Processes

Enzyme isolation and purification are complex processes, and generally rely on various techniques that are sequentially applied to achieve the required purity levels (61). Among the methods that have been developed for this purpose, precipitation, aqueous two-phase partitioning and membrane fractionation have been the most commonly used.

### Precipitation

Proteins, including enzymes, can be precipitated through the disruption of their conformations, which can be achieved through pH shift, ionic strength and/or the addition of water-miscible solvents. Shifting the pH of the medium can either cause protonation of certain groups in the protein (by decreasing the pH), dissociation of protons (by increasing the pH), or charge neutralization (i.e., net electrical charge of the protein in solution is canceled through an equal number of opposite charges) (62). The latter state refers to the isoelectric point in which the protein has the lowest solubility. Precipitation at isoelectric point is widely utilized to recover proteins (63–68), however, it is not usually applied for enzyme isolation due to irreversible denaturation which is the greatest disadvantage of isoelectric point precipitation (69).

The disruption of protein (or enzyme) macro-structures can also be achieved through the addition of significantly high amounts of salt (such as sodium chloride, ammonium sulfate, and sodium sulfate) or through the addition of organic solvents, such as methanol, ethanol, butanol and acetone (62). The large amounts of salt cause protein precipitation through the “salting out” effect. This approach can be used to recover enzymes. However, the salts need to be removed through desalting processes such as dialysis, gel filtration or ion exchange chromatography.

Precipitation through the addition of water-miscible solvents is generally attributed to the competition of protein and the solvent’s polar groups with water (62). If solvent-based precipitation techniques are chosen for enzyme recovery, care has to be taken to extract the enzymes at low temperatures as organic solvents are well known to denature enzymes even at cold temperatures (70).

### Partition Through Aqueous Two-Phase Systems

Aqueous two-phase partitioning is a one-step, liquid–liquid purification approach based on affinity partitioning and is generally achieved by mixing polymer/polymer (typically polyethylene glycol or dextran) or polymer/salt (such as phosphate, sulfate, or citrate) with water (71). In general, polymer/salt based aqueous two-phase systems (ATPS) are preferred due to their lower costs and lower viscosity compared to polymer/polymer based ATPS (72).

In downstream processing, ATPS represents the initial isolation and purification step, which is achieved by the partial elimination of impurities and the reduction of the working volume (73). The simplicity and selectivity of the ATPS process combined with the low cost and the non-toxic nature of phase forming compounds make the large-scale implementation of ATPS possible (72).

Aqueous two-phase systems has been reported to efficiently recover enzymes from fish wastes such as farmed giant catfish viscera (74, 75) and hybrid catfish viscera (76). Despite the promising potential of ATPS to purify enzymes instead of the conventional salt precipitation techniques, some challenges associated with ATPS remain. Firstly, the enzymes still need to be separated from the phase forming reagents after the partition is completed. Secondly, ATPS in industrial settings uses large quantities of chemicals and therefore, sustainable approaches to reuse and/or recycle these chemicals are still needed. Lastly, the use of inorganic salts creates both disposal and environmental issues therefore, new ATPS processes based on biodegradable and/or volatile reagents as alternate to inorganic salts need to developed and optimized (72).

### Membrane Fractionation

Membrane ultrafiltration is a well-established separation process which allows for both purification and concentration (77, 78). Ultrafiltration has routinely been used for the industrial separation and purification of enzymes produced through fermentation (79). The main advantages of ultrafiltration compared to conventional enzyme separation systems is the improved activity recovery (79) as well as the minimum denaturation and/or degradation of bio-products (since ultrafiltration operates under relatively gentle conditions in terms of temperature and pressure) (80). The major disadvantage of ultrafiltration, however, is the membrane clogging due to the precipitates formed by the final product (81).

Despite the advantages of ultrafiltration, only a few research studies have investigated this membrane-based process for the isolation and separation of fish proteolytic enzymes (82, 83). The majority of studies focused on applying ultrafiltration for the fractionation of fish protein hydrolyzates into bioactive peptide fractions.

## Potential Industrial Applications of Fish-Derived Cold-Adapted Proteases

Several potential biotechnological and industrial applications have been suggested for cold-adapted enzymes which have been extensively reviewed elsewhere (22, 23, 26). Among the potential applications, two major industries have been suggested for cold-adapted proteases; laundry detergents and food industries. For instance, in the laundry detergent industry, cold-adapted alkaline proteases, which are active at low temperatures and high pHs, can be used as detergent additives instead of chemical agents or mesophilic proteases that are easily denatured at those harsh conditions (84). In the food industry and due to their high catalytic activity at low temperatures and their instability at elevated temperatures (85), cold-adapted proteases can be used:

(i) In the production of fish protein hydrolyzates and flavoring compounds. In this respect, cold-adapted proteases have been reported to produce fish protein hydrolyzates with acceptable odor in addition to reducing the bitterness and favoring the release of small-size peptides with flavor-forming characteristics (86),
(ii) In the extraction of target ingredients (such as pigments and fish oils). For example, cold-adapted proteases have been shown to enhance the recovery of pigments (i.e., carotene and astaxanthin) from shellfish wastes (87, 88),
(iii) As meat tenderizing agents (89), and
(iV) As rennet substitutes (89).

Other potential industries where cold-adapted fish-derived proteases can find applications include the leather and textile industries [in which cold-adapted proteases can replace harmful chemical reagents (90)] as well as the environmental remediation industry, especially for the digestion of protein-rich solid wastes and liquid effluents in cold weather conditions (91).

Despite the numerous possible applications of cold-adapted enzymes, only a small number have been tested in a real industrial setting. For instance, β-galactosidase, methylesterase, polygalacturonase, and glycogen branching enzyme have been assessed, respectively, in the hydrolysis of lactose present in milk, in fruit firming, in juice processing, and in bread production (85). So far, no large-scale trials have been performed for any suggested industrial applications related to cold-adapted proteases.

## Opportunities of Upcycling Fisheries and Aquaculture Wastes Using Fish-Derived Cold-Adapted Proteases

**FIGURE 1:**
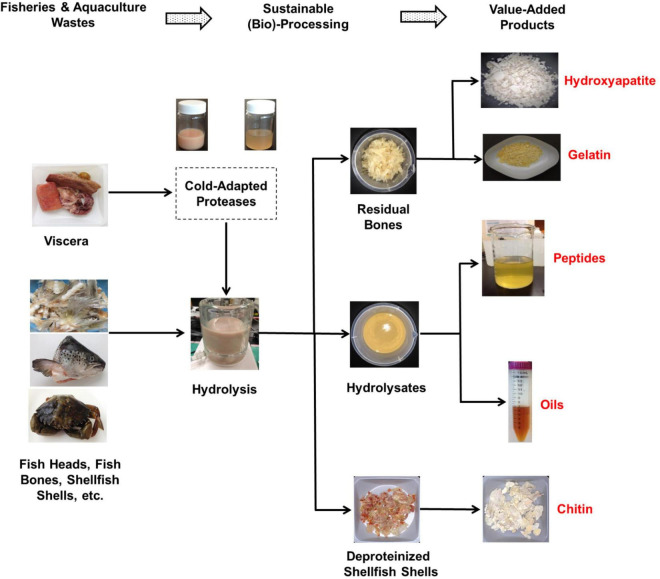
A closed-loop, cost-effective, and environmentally sustainable process for upcycling fisheries and aquaculture wastes using fish- and/or shellfish-derived cold-adapted proteases.

The high specific activity at low temperatures combined with the decreased thermal stability of cold-adapted fish-derived proteases are highly sought after characteristics in various industrial applications (89, 92). It is well known that processing at low temperatures reduces and/or eliminates undesirable chemical reactions, such as oxidation. When cold-adapted acidic proteases are used, the low pH value of the medium significantly lowers the risk of spoilage since acidic conditions inhibit the proliferation of most pathogens (14). Considering these advantageous characteristics, a closed-loop, cost-effective and environmentally sustainable process for upcycling fisheries and aquaculture wastes using cold-water fish-derived cold-adapted proteases can be proposed (as depicted in Figure 1). In this process, cold-adapted endogenous proteolytic enzymes recovered from viscera of cold-water fish species can be utilized as natural biocatalysts to obtain fish hydrolyzates from fish wastes through enzymatic hydrolysis at low temperatures. The produced fish hydrolyzates can further be separated into a number of value-added products such bioactive peptides and omega-3 rich lipids (Figure 1). The bone residues obtained at the end of the hydrolysis (Figure 1) can be converted into either gelatin, which is a multifunctional proteins ingredient in the food industry (93–95) or hydroxyapatite, which is a bioactive and biocompatible product that has a wide range of medical, dental and agricultural applications (96).

Shrimps, crabs, and lobsters are popular seafood around the world (12). The processing of these shellfish generates significant amounts of wastes, in the order of 40–50% of the total mass (97). The proposed process can also be applied in the valorization of crustacean wastes (86, 98). In this regard, shellfish wastes can be hydrolyzed using cold-adapted proteases from cold-water fish species (and/or crustaceans). The enzymatic hydrolysis process for shellfish wastes produces protein hydrolyzates, oils and deproteinized shells (Figure 1). The shells can further be processed into chitin (and/or chitosan) which is a biocompatible, non-toxic and biodegradable polymer with a range of usable applications (11).

## Conclusion and Future Perspective

Research and development on seafood waste valorization has been increasing in the last few years. However, the industrial and commercial implementations of the developed seafood waste valorization approaches are limited in comparison to the large number of research studies that have been carried out and the patents that have been awarded. Currently, rendering seafood waste into fishmeal is still the preferred valorization route, with an annual production of about 5 million tons (99, 100). The present review shows that the recovery of cold-adapted proteases from cold-water fish species can open new opportunities for the development of sustainable, efficient, and cost-effective processes for the valorization of fisheries and aquaculture wastes into more valuable biomolecules (i.e., proteins, peptides, oils, polysaccharides, and hydroxyapatite) compared to fishmeal. However, the availability and variability of fish wastes, remain the most challenging issues for the commercial scale success of this process.

The future perspective of this research topic should center on (i) assessing whether the efficiency of fish-derived cold-adapted proteases in recovering value added products exceeds that of commercial proteases, which could in turn justify and increase the chances for the large-scale implementation of the process, (ii) the isolation and purification of the most promising cold-adapted fish-derived protease using eco-friendly, economic and yet effective extraction processes (such as ATPS) and (iii) the production of the most promising cold-adapted fish-derived protease using genetic engineering and recombinant expression tools.

## Author Contributions

The author confirms being the sole contributor of this work and has approved it for publication.

## Conflict of Interest

The author declares that the research was conducted in the absence of any commercial or financial relationships that could be construed as a potential conflict of interest.

## Publisher’s Note

All claims expressed in this article are solely those of the authors and do not necessarily represent those of their affiliated organizations, or those of the publisher, the editors and the reviewers. Any product that may be evaluated in this article, or claim that may be made by its manufacturer, is not guaranteed or endorsed by the publisher.
